# Dual-Frequency Polarized Reconfigurable Terahertz Antenna Based on Graphene Metasurface and TOPAS

**DOI:** 10.3390/mi12091088

**Published:** 2021-09-09

**Authors:** Jinnan Zhang, Shijie Tao, Xin Yan, Xia Zhang, Jinxuan Guo, Zhiqiang Wen

**Affiliations:** State Key Laboratory of Information Photonics and Optional Communications, Beijing University of Posts and Telecommunications, Beijing 100876, China; zhangjinnan@bupt.edu.cn (J.Z.); taoshijie@bupt.edu.cn (S.T.); xzhang@bupt.edu.cn (X.Z.); guojinxuan@bupt.edu.cn (J.G.); 2020141259@bupt.edu.cn (Z.W.)

**Keywords:** polarization conversion meta-surface, graphene, antenna, terahertz, TOPAS, dual-controlled

## Abstract

A hybrid dual-frequency polarized reconfigurable terahertz antenna is designed and studied. Graphene and TOPAS are employed as the polarization conversion metasurface and dielectric substrate, respectively, enabling tunable polarization conversion and circular polarization. TOPAS is a good substrate material for broadband THz components due to its low absorption. By adjusting the chemical potential of graphene between 0 eV and 0.5 eV, the polarization state in the band of 1 THz (0.76–1.02 THz) and 2.5 THz (2.43–2.6 THz) can be reconstructed. Thanks to the multilayer graphene structure and low absorption TOPAS, the graphene metasurface exhibits a broad bandwidth of 0.26 and 0.17 THz, respectively, in the band of 1 THz and 2.5 THz. The working state of the circularly polarized antenna and linearly polarized antenna can be switched in the bands around 1 THz (0.7–0.75 THz, 0.96–1.04 THz) and 2.5 THz (2.42–2.52 THz), respectively, without changing the physical geometry. Moreover, the graphene antenna, metasurface, and hybrid structure are tested, respectively, to verify that the components do not interfere with each other in performance. The hybrid antenna shows great potential in tunable terahertz devices and related applications.

## 1. Introduction

Terahertz (THz) technology shows bright application prospects in telecommunications [1], imaging, and sensitive detections [2]. As one of the key THz devices, polarized reconfigurable antenna can effectively reduce signal loss caused by polarization mismatch and resist multipath effects, which has attracted increasing attention in recent years. Traditional antennas’ polarization transformation methods include birefringent materials, crystals, optical gratings [3], controlling the on-off of PIN diode [4], and rotating converters to achieve different polarization states [5], which have limitations such as high loss, large volume, and requirements to change the physical structure. Recently, polarization conversion metasurfaces (PCM) in terahertz bands have become a new research hotspot. Meta-material (MM) and meta-surfaces (MS) have more lightweight structures and better performances. For these reasons, MMs have been widely used in micro-nano devices including sensors [6], absorbers [7,8], and antennas [9,10]. Similarly, MMs and MS have been widely used as a new method for changing the polarization state, such as linear-circular [11], linear-linear [12], and circular -circular [13] converters. However, these previously designed structures lack tunability. Therefore, peoples’ research focuses have shifted to the realization of adjustable and switchable MS [14,15].

Graphene is a two-dimension material built up by carbon atoms in a honeycomb lattice [16]. The Fermi energy levels of graphene can be altered by using chemical doping or electrostatic gating [17,18], and the electrical conductivity of graphene is correspondingly changed, which makes it a promising candidate for the design of tunable devices. Therefore, graphene-based PCM is expected to achieve good tunability [19,20,21]. However, graphene PCM [22,23,24] has single polarization properties, which is still limited in functionality. One promising way is to combine antenna with graphene PCM to expand the polarization conversion functionality, which is expected to enable tunable linear polarization and circular polarization characteristics at the same working frequency band. Moreover, multi-band devices are particularly promising in biomedicine [25], terahertz imaging [26], molecular material detection [27] and other advanced fields, but most miniaturized reconfigurable resonant antennas [28,29] have only one frequency band and cannot work in dual frequency bands with different polarization states at the same time.

In this work, a hybrid dual-frequency polarizable polarized reconfigurable THz antenna is designed and studied. Graphene is employed as the PCM material to achieve reconstruction of the polarization state. TOPAS [30,31] is selected as the flexible dielectric substrate, which is a good substrate material for broadband THz components due to its low absorption. By adjusting the chemical potential of graphene between 0 eV and 0.5 eV, the polarization state in the band of 1 THz (0.76–1.02 THz) and 2.5 THz (2.43–2.6 THz) can be reconstructed. Combined with the terahertz antenna, the working state of the circularly polarized antenna and linearly polarized antenna can be switched in the bands around 1 THz (0.7–0.75 THz, 0.96–1.04 THz) and 2.5 THz (2.42–2.52 THz) without changing the physical geometry. This combination can easily transform the linearly polarized THz antenna into a tunable, multi-state THz antenna, and also provide a way for the design of traditional circularly polarized antennas.

## 2. Materials and Design

### 2.1. Graphene Material

The conductivity of graphene is provided by Kubo’s equation [32], which is determined by both intra-band and inter-band transitions.

(1) $$\sigma_{S}=\sigma_{intra}+\sigma_{inter}$$

(2) $$\sigma_{intra}=-j\frac{e^{2}K_{B}T}{\pi \hbar^{2}\left(\omega -j2\Gamma \right)}\left(\frac{E_{F}}{k_{B}T}+2ln\left(e^{-E_{F}/k_{B}T}+1\right)\right)$$

(3) $$\sigma_{inter}=\frac{-je^{2}}{4\pi \hbar }\ln\left(\frac{2\left|E_{F}\right|-\left(\omega -j2\Gamma \right)\hbar }{2\left|E_{F}\right|+\left(\omega -j2\Gamma \right)\hbar }\right)$$

In these equations, *e* is the charge of an electron,$\;k_{B}\;$is Boltzmann’s constant,$\;E_{F}\;$is the Fermi energy, ω=2πf is the angular frequency, and ℏ=h/2π is a reduced Planck’s constant. In the simulations, *T* is the environmental temperature, which is fixed at 300 K, Γ=1/2τ is the phenomenological scattering rate, where τ=0.5 ps is the electron-phonon relaxation time. The main advantage of graphene is that its surface conductivity can be tuned by changing the Fermi energy. By applying a transverse electric field through a bias gated structure, $E_{F}$ can be adjusted over a wide range (between ±1.0 eV), so the conductivity of graphene can be controlled by a DC bias voltage. An approximate closed-form expression between Fermi energy $E_{F}\;$and bias voltage *V_g_* is given by Ref [33].

(4) $$E_{F}\approx \hbar \nu_{f}\sqrt{\frac{\pi \varepsilon_{r}\varepsilon_{0}V_{g}}{et_{s}}}$$

In Equation (4), $\nu_{f}$ is the Fermi velocity, which is fixed at $1.1\times 10^{6}$ m/s, *V_g_* is the bias voltage, which can be artificially controlled, $\varepsilon_{0}$ and $\varepsilon_{r}$ are the permittivity of the vacuum and dielectric, and $t_{s}$ is the thickness of the insulating spacer. In summary, Equations (1)–(4) provide an effective solution to dynamically control the polarization state of terahertz wave by bias voltage.

### 2.2. TOPAS Material

The full name of TOPAS polymer is Topas Cyclic Olefin Copolymer (Topas COC) [31]. Cyclic olefin copolymers (COC) are a new class of optical thermoplastics that have a number of attractive properties including high optical transmission, low birefringence, and low moisture uptake; they are copolymers of ethylene and a cyclic olefin such as norbornene or cyclopentene [34]. TOPAS is one of the injection moldable COCs available commercially [35], which has potential applications in THz wave guiding due to the very low absorption [31]. Broadband THz spectroscopy on a 3.2 mm thick sample shows a refraction index between 1.52 and 1.53 and absorption <$\;3{\;\mathrm{cm}}^{-1}$ across the THz band [36]. TOPAS also provides excellent resistance to most acidic solvents; these properties make it an ideal choice for wideband THz optical components and wideband THz spectral substrates, including window materials and waveguides. Up to now, there are many pieces of research on devices using TOPAS materials in the field of THz, including the absorber using TOPAS and VO2 [37] and the polarization converter using TOPAS [38]; both have significant improvements in operating bandwidth.

### 2.3. PCM Unit

**Figure 1:**
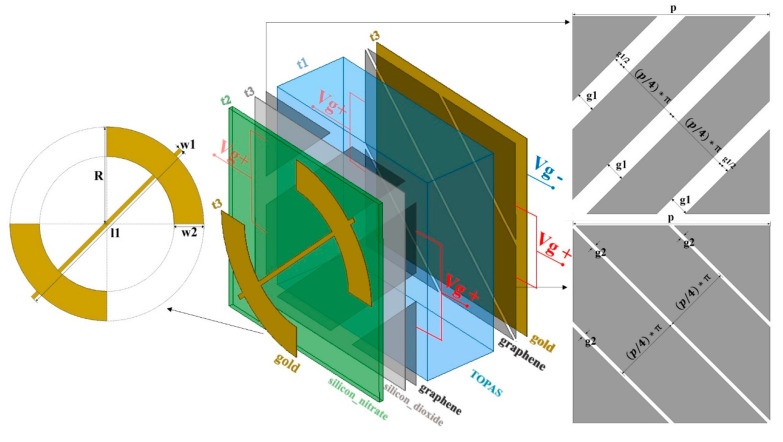
Structure of PCM unit.

The schematic of the PCM unit is shown in Figure 1, which is a reflective structure consisting of seven layers. From front to back are: gold pattern layer, silicon nitrate layer (relative dielectric constant is 7), silicon dioxide layer (relative dielectric constant is 4), graphene layer 1, TOPAS layer (loss tangent is about 0.00007 [38], relative dielectric permittivity of 2.35 [37]), graphene layer 2, and a gold reflection layer. Optimal geometrical parameters of the PCM unit are shown in Table 1.

The main purpose of adjusting the parameters is to make the PCM unit match the operating frequency of the antenna, and achieve higher performance (higher polarization conversion ratio (PCR) and wider operating bandwidth) of the PCM unit in “3.1 PCM unit’s performance”. Ansys High-Frequency Structure Simulator (HFSS) Commercial software was used for the optimization of the parameters. Specifically, the influence of individual parameters, t1, t2 and p will affect the distance between the two peak frequency points of PCR, w1 and w2 will affect the position and height of the right peak of PCR, and other parameters will affect the height of two peaks of PCR.

As shown in Figure 1, two graphene layers with slits along the diagonal directions are located, respectively, above and below the TOPAS layer. It is worth mentioning that the chemical vapor deposition (CVD) [39] process is generally used in the synthesis process to achieve the multilayer graphene-dielectric structure [40]. The graphene layers were grown on copper foil by CVD and then transferred to TOPAS layers. The slits patterns on the graphene layers were etched by UV laser, which had a wavelength of 355 nm and a resolution of 20 µm [41]. Epitaxial graphene on SiC [42] or chemical reduction of exfoliated graphite oxide [43] can also be considered to fabricate graphene sheets. Additionally, the gold patterns on graphene-based electronics are fabricated using electron beam lithography (EBL) [44] or a focused Ion beam (FIB); now, FIB systems offering resolution down to 10–15 nm are commercially available [45].

In practice, we recommend using the external DC bias voltage Vg to adjust the Fermi energy of the graphene layer; the voltage difference between Vg+ and Vg− is Vg. The dynamic polarization manipulation of the PCM unit is mainly achieved by adjusting the birefringence of the multifunctional metamaterial through electrically shifting the Fermi energy of both graphene layers. The Fermi energy levels of the two graphene layers were simultaneously adjusted, and state 1 was set at 0.0 eV, state 2 was set at 0.5 eV.

### 2.4. Dual-Band THz Antenna

**Figure 2:**
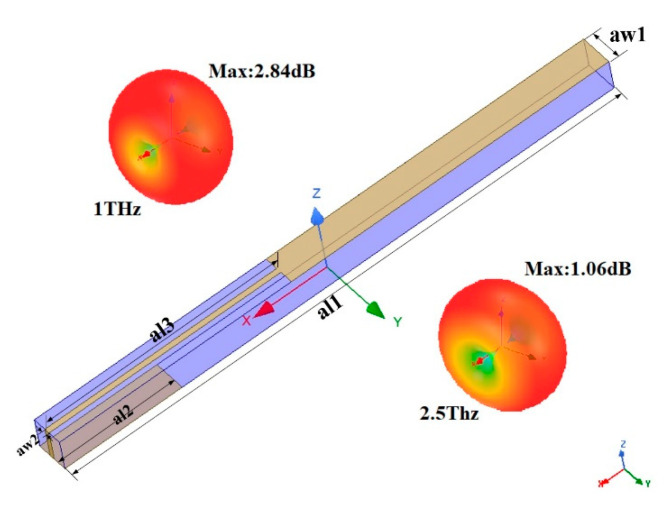
Structure of a dual-band THz antenna. 3D radiation pattern of the antenna at 1 and 2.5 THz.

The structure of the designed antenna is shown in Figure 2, which consists of two layers and is printed on the Rogers 4350 (relative permittivity is 3.66) substrate. The top layer is composed of rectangular metal patches and metal feeders, and the bottom layer contains a rectangular metal. Optimal geometrical parameters of the antenna are shown in Table 2.

### 2.5. Antenna-PCM Hybrid Structure

**Figure 3:**
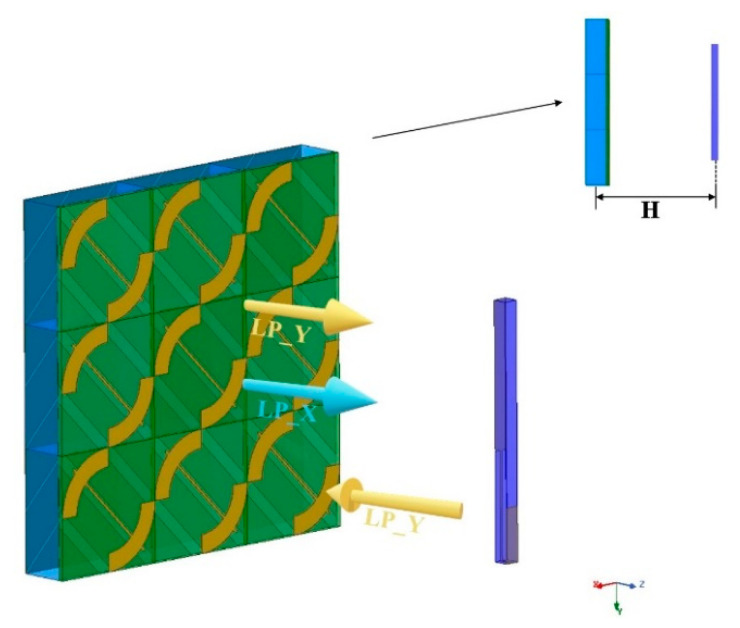
Polarization conversion diagram of the antenna-PCM hybrid structure.

Figure 3 shows the 3 × 3 PCM arrangement and the polarization conversion diagram of the antenna-PCM hybrid structure. It is found that the distance between the center of the antenna and PCM array affects the ellipse and phase difference of the structure. In this work, an optimal distance of 200 μm (H in Figure 3) is employed. In this way, the Y polarization wave is vertically incident upon the PCM array. By changing the chemical potential of graphene, the polarization state of the reflected wave could be switched between X-polarization and Y-polarization.

### 2.6. Simulation Setup

Ansys HFSS commercial software is used for the simulation of the proposed structure. The THz wave impinges on the proposed device from the air. Periodic linked boundary conditions (primary and secondary) are adopted in the x- and y-directions and Floquet port excitation in the z-direction. Additionally, in a hybrid structure test, the antenna serves as the signal source with an incentive source of Lumped port. To better describe a graphene film, the graphene layer is considered as a two-dimensional conductive surface with the impedance boundary of resistance and reactance in the software simulations. TOPAS material is a three-dimensional material with a specific relative permittivity and loss tangent. In the analysis setup, the maximum number of passes was set to 18, and delta S was 0.02.

## 3. Results and Discussions

### 3.1. PCM Unit’s Performance

**Figure 4:**
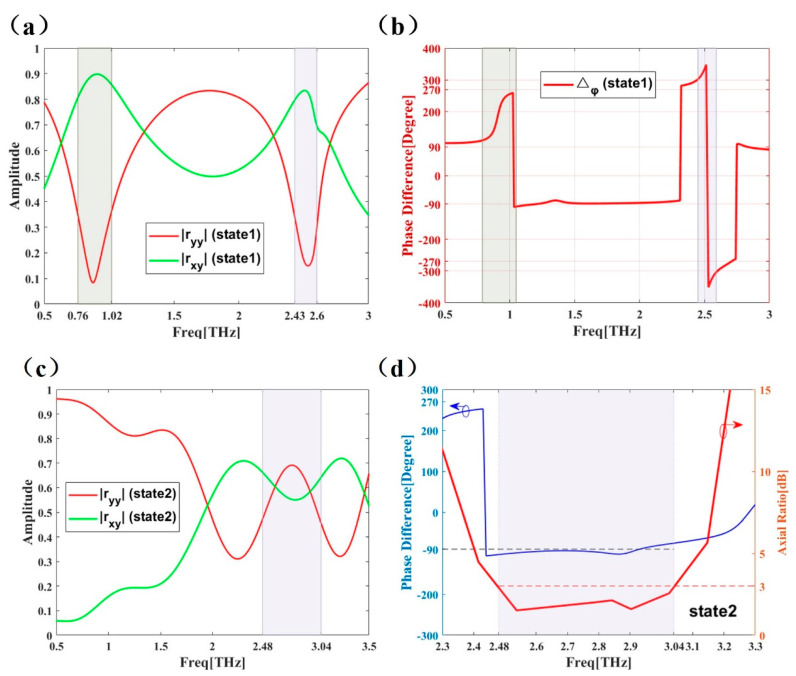
The $\left|r_{\mathrm{yy}}\right|$ and $\left|r_{\mathrm{xy}}\right|$ amplitude of the PCM unit at state 1 (0.0 eV) (**a**) and state 2 (0.5 eV) (**c**). The phase difference $\Delta \phi_{\mathrm{xy}}$ of PCM unit’s $\left|r_{\mathrm{yy}}\right|$ and $\left|r_{\mathrm{xy}}\right|$ at state 1 (0.0 eV) (**b**) and state 2 (0.5 eV) (**d**). Axial ratio of PCM unit at state 2 (0.5 eV) (**d**).

The performance of the PCM unit near 1 THz and 2.5 THz under a Y-polarized incident wave is shown in Figure 4. Here, $\left|r_{\mathrm{yy}}\right|$ is the co-polarized reflection coefficient and $\left|r_{\mathrm{xy}}\right|$ is the cross-polarized reflection coefficient. Figure 4a shows the data for state 1, corresponding to a graphene Fermi energy of 0 eV. In the 0.76–1.02 and 2.43–2.6 THz bands, $\left|r_{\mathrm{yy}}\right|\;$is small but $\left|r_{\mathrm{xy}}\right|$ is high, indicating that the incident Y-polarized wave is converted into an X-polarized wave after being reflected by PCM. The amplitude of the X polarization wave is about 80% of that of the incident Y polarization wave, while the amplitude of the reflected Y polarization wave is reduced to less than 30%. Figure 4b shows the phase and phase difference $\Delta \phi_{\mathrm{xy}}$ of the co-polarized and cross-polarized components in state 1. The phases of the low frequency band are around ±90° and 270°, while that of the high frequency band is around ±270°. According to the classical electromagnetic field theory, the electric field directions of the reflected X-polarized wave and the incident Y-polarized wave are orthogonal. For state 2, corresponding to a graphene Fermi energy of 0.5 eV, the amplitude of converted waves is similar and is around 60% of that of the incident wave amplitude in the 2.48–3.04 THz band, as shown in Figure 4c. In Figure 4d, $\Delta \phi_{\mathrm{xy}}\;$is around −90° and the axial ratio is less than 3dB, indicating that the reflected electromagnetic wave is right-handed circularly polarized (RCP).

From Figure 4, it is found that the polarization conversion characteristics in state 1 are different from that in state 2. In the 1 THz band, a Y polarization wave can be converted into X polarization wave in state 1, but no conversion occurs in state 2. In the 2.5 THz band, Y polarization wave can be converted to X polarization wave in state 1, while in state 2 the reflected wave is converted into circularly polarized waves.

**Figure 5:**
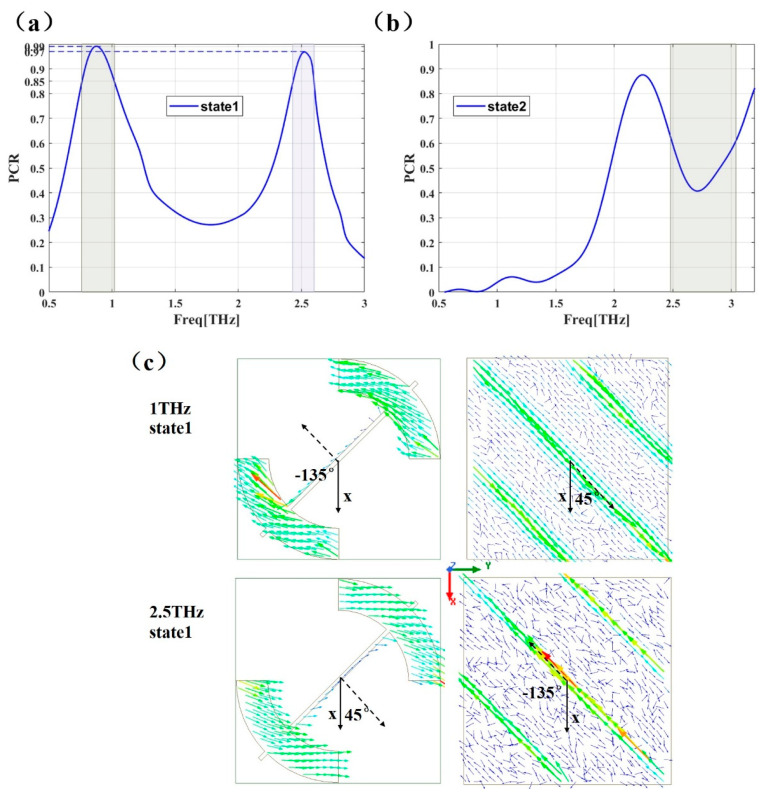
Polarization conversion ratio of PCM unit at state 1 (0.0 eV) (**a**) and state 2 (0.5 eV) (**b**). (**c**): The surface current distribution on the top layer (1st column) and the bottom layer (2nd column) of the PCM unit in state 1, the dashed arrows represent the direction of surface current.

PCR is described by Equation (5), where $\left|r_{\mathrm{xy}}\right|$ and $\left|r_{\mathrm{yy}}\right|$ represent the cross and co polarization coefficient, respectively. A PCR close to 1 means that Y-line polarized wave is converted to X-line polarized wave after being reflected by the surface. A PCR close to 0 means that a Y-polarized wave is still Y-polarized. A PCR close to 0.5 means that X-polarization and Y-polarization wave possess the same amplitude. As is shown in Figure 5, for state 1; the PCR has two peak values of 0.99 and 0.97 in the bands of 0.76–1.02 THz (0.26 THz bandwidth) and 2.43–2.6 THz (0.17 THz bandwidth), respectively. For state 2, the PCR is lower than 0.1 in the 0.76–1.02 THz band and around 0.5 in the 2.48–3.04 THz (0.17 THz bandwidth) band, which was consistent with the previous discussions about Figure 4.

(5) $$\mathrm{PCR}=\frac{|r_{xy}{|}^{2}}{\left|r_{xy}{|}^{2}+\right|r_{yy}{|}^{2}}$$

The bandwidth of a converter may generally be extended by stacking multilayer structures [46], applying plasmon hybridization [47], or by using various plasmon resonances [48]. Here, two-layer graphene structure and TOPAS are employed to increase the bandwidth. As has been reported, broadband THz spectroscopy of a 3.2 mm thick TOPAS sample shows an index of refraction between 1.52 and 1.53 and absorption < 3 cm^−1^ across the THz band [36], which make it ideal for broadband THz components. Our PCM unit shows a broad operating bandwidth of 0.26 THz and 0.17 THz in the 1 THz and 2.5 THz bands, respectively, much higher than the previously reported results. At the same time, some reconfigurable hybrid structures have only one frequency band and cannot work in dual frequency bands with different polarization states at the same time, the proposed PCM has advantages over it. main parameters of the proposed design and similar works in references are listed in Table 3 for comparisons

Next, in order to investigate the working principle behind the PCM unit, we then simulated the surface current distributions on both the bottom and top metal layers at the resonance frequency of 1.0 THz and 2.5 THz in state1 and under an y-polarized incident wave, the results are given in Figure 5c. It can be seen that the strongest current density on the bottom layer is along the slots (on the second graphene layer) on the secondary diagonal having 45° (1.0 THz) and −135° (2.5 THz) angle with X-axis, the surface currents on the two layers at 1.0 THz and 2.5 THz are opposite to each other. In other words, they form a current loop in the intermediate dielectric substrate, and this equivalent circulating current verifies the magnetic resonance of the device [52]. Such resonance is essential for achieving high efficiency and wide bandwidth, the induced magnetic response is always 45° relative to the incident polarized wave, the magnetic field can be decomposed two vertical components H_x_ and H_y_. H_x_ is perpendicular to the incident electric field E, there is no cross-coupling because the incident and reflected magnetic fields are in the same direction [53]. H_y_ parallel to the incident electric field E, it produces an induced electric field perpendicular to the incident electric field E, so the incident wave can be converted to orthogonal polarization [54,55].

### 3.2. Dual-Band THz Antenna’s Performance

**Figure 6:**
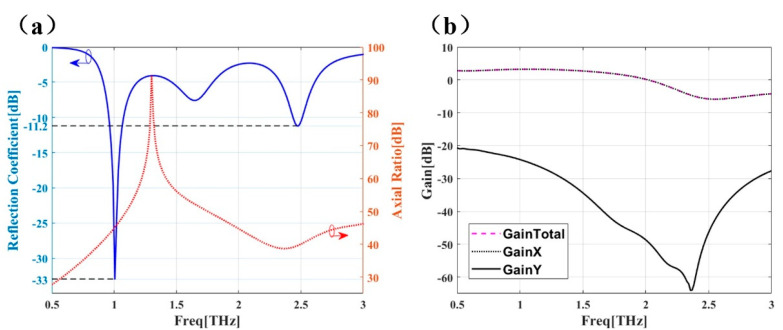
(**a**) Reflectance coefficient and axial ratio of dual-band THz antenna. (**b**) Gain in X and Y directions and total gain of dual-band THz antenna.

Now we turn to study the performance of dual-band THz antennas. The blue line in Figure 6a presents the reflection coefficient of the antenna, suggesting a dual-band THz antenna with two resonant frequencies (1 THz and 2.5 THz), which matches the operating bandwidth of the PCM. The red line in Figure 6a suggests that the axial ratio of the antenna remains above 25 dB in a broad range of 0.5–3 THz, indicating that the electromagnetic wave generated by the antenna is polarized. In Figure 6b, it can be seen that the gain in the X direction and the total gain are approximately the same, but the gain in the Y direction is very low. This means that the antenna can emit polarization waves in the X direction. Hence, the hybrid structure based on antenna and PCM array shown in in Figure 3 is employed.

### 3.3. Performance of the Antenna-PCM Hybrid Structure

**Figure 7:**
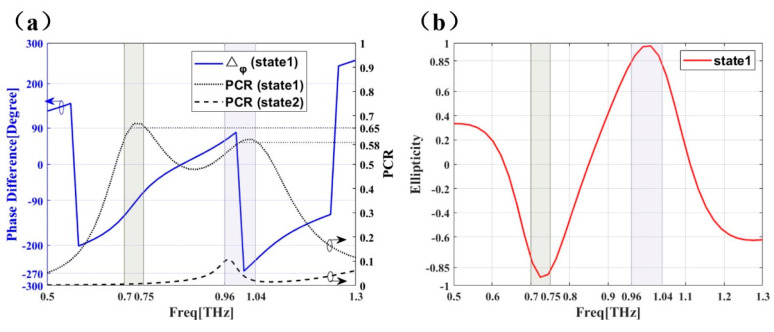

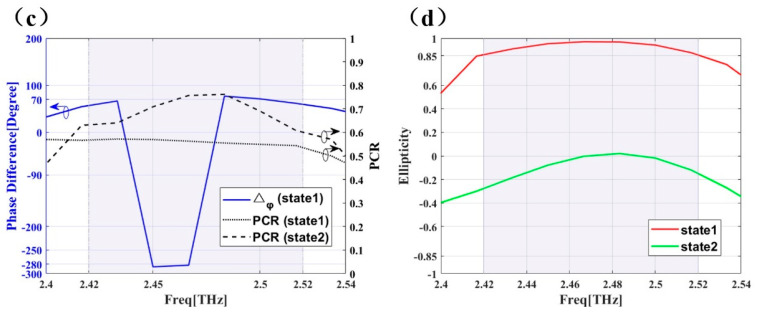
$\Delta \phi_{\mathrm{xy}}$ and PCR of antenna-PCM composite structure in 1 THz (**a**) and 2.5 THz (**c**), Normalized ellipticity of antenna-PCM combination structure in 1 THz (**b**) and 2.5 THz (**d**) at state 1 (0.0 eV) and state 2 (0.5 eV).

The performance of the antenna-PCM hybrid structure is show in Figure 7. We focus on the performance near 1 THz and 2.5 THz. Here, the normalized ellipticity (*χ*), which can judge the degree of circular polarization of each frequency point swept [38], is used to verify the circular polarization characteristics of the device. χ can be described as follows:

(6) $$\chi =\frac{2\left|r_{\mathrm{yy}}\right|\left|r_{\mathrm{xy}}\right|\sin\Delta \phi_{\mathrm{xy}}}{\left|r_{\mathrm{yy}}{|}^{2}+\right|r_{\mathrm{xy}}{|}^{2}}$$

where $\left|r_{\mathrm{xy}}\right|$ and $\left|r_{\mathrm{yy}}\right|$ represent the cross and co polarization coefficient, and represents the phase difference. *χ* close to 1 represents the left circular polarization (LCP), and close to −1 represents RCP, as a circularly polarized wave needs to meet the two requirements of equal polarization wave amplitude and phase difference.

In state 1, the PCR near 1 THz is shown in Figure 7a, which is divided into two frequency bands: (1) PCR in the 0.7–0.75 THz frequency band is around 0.65, indicating that the co and cross-polarization reflection coefficients are similar at this time. The blue line ($\Delta \phi_{\mathrm{xy}}$) indicates that the phase difference is around −90°, which means that in this frequency band RCP waves would be generated. Meanwhile, χ of this band is close to −1, as shown in Figure 7b. (2) PCR in the 0.96–1.04 THz frequency band is around 0.58, and $\Delta \phi_{\mathrm{xy}}$ is around 90° and −270°, indicating that LCP waves would be generated in this frequency band. Meanwhile, *χ* is close to 1, as shown in Figure 7b. In state 2, PCR near 1 THz is below 0.1, indicating that the Y-polarization wave of the antenna is not converted. This is due to the high PCR property of PCM at 1 THz (Figure 5a), which converts the Y-polarization wave of the antenna into the X-polarization wave with same amplitude. At the same time, it combines with the antenna’s own Y-polarization wave to form the circular polarization. In state 2 the low PCR characteristic of PCM leads to the low PCR of the hybrid structure, which is dominated by Y-polarization waves. This also demonstrates that the antenna and PCM array will not interfere with each other in performance.

A PCR near 2.5 THz in state 1 is shown in Figure 7c. A PCR between 2.42 THz and 2.52 THz is around 0.55, which indicates that the reflection coefficients of co-polarization and cross-polarization are similar. $\Delta \phi_{\mathrm{xy}}$ is at 70° and −280°, which indicates that LCP waves will be generated in this frequency band. Meanwhile, χ of the frequency band is close to 1, as shown in Figure 7d. Similar to the case of 1 THz band, the circular polarization characteristic of the hybrid structure is the result of the combination of the antenna Y wave and PCM reflected wave. In state 2, PCR near 2.5 THz is around 0.6, and *χ* in Figure 7d is around −0.2, indicating that the reflected wave is not LCP or RCP waves. This is because that the Y and X polarized waves of the hybrid structure are not the same. As shown in Figure 5b, the amplitude of Y polarized waves of the hybrid structure is larger, and the phase difference does not meet the orthogonality.

## 4. Conclusions

In summary, a hybrid dual-frequency polarized reconfigurable THz antenna is designed and studied. The employment of graphene metasurface and TOPAS enables tunable polarization conversion and circular polarization. By electrically shifting the Fermi energy of both graphene layers, the polarization state of the two bands can be changed without reconstructing the structure. Besides, the results demonstrate that the antenna and PCM array will not interfere with each other in performance. The proposed device has great potential in tunable THz systems.

## Figures and Tables

**Table 1:** Optimal geometric parameters of the PCM unit.

Parameter	Length (um)	Parameter	Length (um)
t1	18.5	w2	15
t2	2	R	49
t3	0.1	p	49
w1	1	g1	5
l1	53	g2	1

**Table 2:** Optimal geometric parameters of the dual-band THz antenna.

Parameter	Length (um)
aw1	6
aw2	1
al1	103
al2	22
al3	44

**Table 3:** Comparison of parameters of similar works in references.

Reference	Tunability	Bandwidth	Centre or Resonance Frequency	Hybrid Structure
[49]	No	3.98 GHz (9.38–13.36 GHz)	11.4 GHz	No
		5.52 GHz (14.84–20.36 GHz)	17.6 GHz	
[50]	No	5.52 GHz (14.84–20.36 GHz)	5.97 GHz	Yes
		1.23 GHz (5.40–6.63 GHz)	6.02 GHz	
[29]	Yes	180 GHz (1.38–1.56 THz)	1.44 THz	Yes
[51]	Yes	28 GHz (1.302–1.33 THz)	1.32 THz	Yes
		80 GHz (1.46–1.54 THz)	1.51 THz	
This paper	Yes	260 GHz (0.76–1.02 THz)	1.0 THz	Yes
		170 GHz (2.43–2.67 THz)	2.5 THz	

## Data Availability

Not applicable.
